# Assessing Self-Awareness through Gaze Agency

**DOI:** 10.1371/journal.pone.0164682

**Published:** 2016-11-03

**Authors:** Regina Gregori Grgič, Sofia Allegra Crespi, Claudio de’Sperati

**Affiliations:** 1 Faculty of Psychology, Vita-Salute San Raffaele University, via Olgettina 58, 20132 Milan, Italy; 2 Laboratory of Action, Perception, Cognition, Vita-Salute San Raffaele University, via Olgettina 58, 20132 Milan, Italy; 3 Experimental Psychology Unit, Division of Neuroscience, IRCCS San Raffaele Scientific Institute, via Olgettina 60, 20132 Milan, Italy; 4 CERMAC, Neuroradiology Department, IRCCS San Raffaele Scientific Institute, via Olgettina 60, 20132 Milan, Italy; Universitatsklinikum Tubingen, GERMANY

## Abstract

We define gaze agency as the awareness of the causal effect of one’s own eye movements in gaze-contingent environments, which might soon become a widespread reality with the diffusion of gaze-operated devices. Here we propose a method for measuring gaze agency based on self-monitoring propensity and sensitivity. In one task, naïf observers watched bouncing balls on a computer monitor with the goal of discovering the cause of concurrently presented beeps, which were generated in real-time by their saccades or by other events (Discovery Task). We manipulated observers’ self-awareness by pre-exposing them to a condition in which beeps depended on gaze direction or by focusing their attention to their own eyes. These manipulations increased propensity to agency discovery. In a second task, which served to monitor agency sensitivity at the sensori-motor level, observers were explicitly asked to detect gaze agency (Detection Task). Both tasks turned out to be well suited to measure both increases and decreases of gaze agency. We did not find evident oculomotor correlates of agency discovery or detection. A strength of our approach is that it probes self-monitoring propensity–difficult to evaluate with traditional tasks based on bodily agency. In addition to putting a lens on this novel cognitive function, measuring gaze agency could reveal subtle self-awareness deficits in pathological conditions and during development.

## Introduction

Eye movements are at the service of vision. However, despite their role in orienting the retina, the mobile eyes do not have only a perceptual function. Sometimes eye movements may act causally on the world. Indeed, babies learn very soon that the other people may react to their gaze [1,2], e.g., mums may smile upon eye contact. Similarly, in monkeys eye contact with conspecifics has a high social valence, and direct fixation may foster aggressive behavior [3]. This causal role of gaze, however, is found essentially in the social domain [4]: soap bubbles do not explode by gazing at them. This basic evolutionary principle may become soon obsolete: it is perhaps just a matter of time before our daily life will be pervasively populated by gaze-operated devices. We might soon discover that the spot on the traffic light is in fact a “gaze button” for a green request, that our preferred videogame can be played with the eyes, or that we can easily browse the museum virtual collection–or the list of our mobile apps–with eye movements. Thus, in parallel with the diffusion of gaze-contingent displays and attention-aware devices, the ability of using the eyes as a full-fledged effector to act on the physical/artificial world will become an increasingly important function which will add up to our behavioral and cognitive repertoire [5,6,7]. It is time to put a lens on this ocular function we are not endowed with at birth. We call it gaze agency.

Very generally, agency refers to the capability of an individual to act causally in the world. For the sake of completeness, the extended designation of gaze agency, as we use in the present study, should be “sense of gaze-operated self-agency in non-social contexts”. It is a “sense” because it refers to the subjective perception of being the one who is performing an action. It refers to the “gaze” because it identifies the effector rather than the achieved effects (in our study eye movements produces an acoustic event). It is “self” because it refers to one’s own agency, not agency of other individuals. Finally, as anticipated, we are not targeting the social gaze but the very peculiar ability to direct the gaze on the physical/technological world in order to operate a change in it. This function of the gaze corresponds to a performative action, as contrasted to exploratory and communicative actions, pertaining respectively to the perceptual and social domains [8].

Studying gaze agency requires to develop a tool or a method to measure it. In the past, the sense of agency has been measured using essentially three different approaches. One method is based on temporal-binding, and exploits the capability of perceiving the temporal unity between an action and a causally linked event [9,10]. Another method is based on sensory attenuation, and exploits the reduction of the sensory intensity of the consequences of an action [11,12,13]. Alternatively, the sense of agency can be directly investigated through scales and questionnaires testing self versus non-self attribution [13,14,15]. In the cited experiments, the subjects’ task was to evaluate the effects of their voluntary body (hand/finger) movements (bodily agency), i.e., they were told in advance what to pay attention to. For example, in a recent study observers were asked to perform hand movements in open-loop conditions while watching on a computer screen the image of their own hand or an alien hand executing the same or a different movement in real time, with the task of discriminating whether the hand presented on the screen was their own hand or not [16]. In another study, Repp & Knoblich [17] distinguished between “active” sensori-motor synchronization and “passive” auditory synchronization in the attempt to tease apart “cognitive” and “perceptual” agency. In their study, observers had to identify when a sequence of acoustic stimuli were driven by their finger tapping or were externally driven. This however prevents spontaneous agency discovery and modifies the natural balance between inward- and outward-directed attention (e.g., [16,18,19,20]). Using gaze instead of bodily movements offers the opportunity to measure agency in a different way because saccades are the most frequent spontaneous motor act, thus an explicit request to perform a given movement and to pay attention to its consequences is not necessary. However, we know almost nothing about gaze agency. Arguably, one reason is that only recent interaction technology endowed the gaze with performative function.

A first step to investigate gaze agency was made by Lindner and colleagues [21], who used the Filehne illusion to assess the matching of visual and oculomotor signals during smooth pursuit eye movements. They were able to show a deficit of visuo-motor matching in those schizophrenic patients exhibiting a defective sense of agency. This finding pointed to a failure of low-level sensori-motor mechanism in charge to compensate the sensory consequences of one’s own movements in schizophrenia [22]. More recently, other studies addressed the relation between saccades and their visual effects [23,24], thus further stressing the importance of proper sensori-motor contingencies in visual perception and action [25]. For example, in a recent study [26] observers were shown two different faces concurrently presented in the left and right sides of the visual field, and a tone paired with a given ocular action (i.e., making a spontaneous rightward or leftward saccade to one of the two faces). In a subsequent test phase, observers were presented two identical faces, preceded by the tone. It was found that the preceding acquisition phase induced an association between the sensory event and the ocular action. The authors noted that “*most adults were unaware of the saccade-effect mapping*, *which is in line with the idea that action-effect acquisition is a low-level*, *fast and automatic process that does not require attention*”. This effect was present in both adults and 12-months infants.

However, there is more to agency than perceptual compensation or automatic associations [27,28,29]. Indeed, a specific failure of explicit self-agency attribution has been reported in those schizophrenic individuals that can misattribute the agent of a hand movement without significant impairments in visuo-motor coordination [30], suggesting that, in general, the sense of agency entails forming a correspondence between intention, action and the results of action [31,32,33]. For a proper sense of agency to be in place, individuals should have self-monitoring capabilities, which, in turns, require a certain degree of self-awareness and internally-directed attention (see [34,35]). In this sense, our study goes beyond the study of Lindner and colleagues, who developed a task tailored to assess retinal slip cancellation–a reflex-like sensori-motor mechanism–through a perceptual illusion [36]. It goes also beyond the study of Verschoor and colleagues, who targeted pre-attentive mechanisms [26]. By contrast, our approach is cognitively-oriented, as it requires observers to understand the causal relation between their own eye movements and external physical events. That is, it focuses on self-awareness.

Here we present a method to probe self-awareness through gaze agency. The method is based on two tasks, a novel, relatively unstructured task aimed at capturing the spontaneous discovery of gaze agency, and a second task in which the capability to perceive gaze agency was structured as a more traditional psychophysical forced-choice detection task. In the first task, called “Discovery Task”, observers watched on the computer screen several moving and bouncing balls, and had to discover the cause of the beeps they were concurrently hearing. In the main condition, a beep was generated whenever the observer made a saccade, while in other conditions beeps were variously generated by other internal or external events (in which case beeps depended on observer’s generic gaze direction or balls behavior, respectively). This unconstrained behavioral task was intended to mimic the natural discovery of the causal relation between two events. We manipulated two factors related to self-awareness: i) previous exposure to a gaze-contingent condition–where the gaze acquires a performative function–intended to help observers to become aware of their own gaze agency capability; ii) a verbal hint to focus on one’s own gaze, which invited observers to regard themselves as causal agents. In both cases the experimental manipulation tended to orient attention towards oneself, whose effects can be measured in terms of discovery rate. By contrast, in the second task, called “Detection Task”, participants had to explicitly tell whether or not the beeps were generated by their eye movements. Thus, this task was more suited to reveal agency sensitivity under focused attentional load than to probe the spontaneous tendency towards internally- or externally-directed attention. The study was conducted on healthy adults, and was intended as a first step towards its application in developmental and clinical investigations. Preliminary results have been presented in abstract form [37].

## Methods

### Participants

Thirty participants (aged between 20 and 29, 8 males, 22 females) volunteered for the experiments. They had normal or corrected-to-normal vision, and were naïve as to the purpose of the study. The experiments have been conducted in accordance with the Declaration of Helsinki. The San Raffaele Ethical Committee approved this study. Before the beginning of the experiment, each participant signed the written informed consent.

### Stimuli and tasks

#### Discovery Task

**Fig 1:**
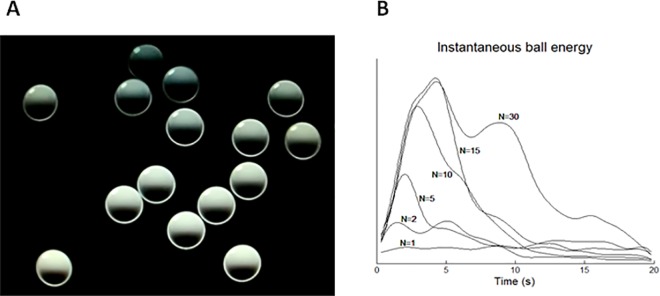
Left panel A, a snapshot of the moving balls display with N = 15. Right panel B, smoothed energy profiles of the balls in the different trials. Energy increases rapidly during the initial part of the trial, when the balls progressively enter the display, whereas it decreases more slowly afterwards, as an effect of the ball bounces. The bounces tend normally to decrease the ball energy, but occasionally they increase it (“squeezing effect”, visible as “bumps” in the curves). N indicates the number of balls in each trial).

Participants seated in front of a computer screen (Dell Trinitron, 21”, framerate: 60 Hz, viewing distance: 57 cm) in a moderately darkened room, with the head restrained by means of a chin rest and a forehead abutment. The visual stimulus consisted of a number of grey billiard-like balls (diameter 2.6 deg) that entered progressively the display from the bottom left corner of the screen. The balls moved pseudo-randomly along a linear trajectory, at constant velocity, bouncing one against the other and against the display’s borders, tending to loose energy at each bounce. The stimulus lasted for 20 seconds (trial duration), during which a sequence of beeps was also generated. Each beep consisted of a 1000 Hz, 40 ms tone, delivered through earphones. The number of balls decreased in each subsequent trial: 30, 15, 10, 5, 2, 1. In the 7^th^ trial, 2 stationary balls were displayed at an eccentricity of 5 deg to the left and the right of the central screen position. **Fig 1A** shows a snapshot of the visual stimulus during the second trial (N = 15). The subjects’ task was to guess what generated the beeps. At the end of each trial observers were requested to report verbally their conjecture regarding the possible cause of the beeps, together with a confidence rating (range 1–5: 1 null confidence, 5 highest confidence). If the response was correct–and with the highest confidence–for two consecutive trials, the remaining trials were skipped and the respective responses automatically considered as correct and with the highest confidence score. By reducing the number of balls in successive trials, we aimed at providing a controlled discovery path in which the likelihood that the ball bounces were the cause of the beeps progressively tended to zero.

The Discovery Task comprised four conditions. In the main Condition (‘Saccades’) the beeps were generated by observer’s saccades. We used a stringent criterion for real-time saccade detection that excluded micro-saccades (eye tangential velocity threshold = 120 deg/s) in order to avoid excessive difficulties to the observers (micro-saccades may go completely unnoticed, [38]). A “refractory period” of 200 ms was added to avoid occasional double-beeps, which again might confound the observers. In this condition an additional 8^th^ trial was administered, identical to the 7^th^ trial, but which was preceded by a pre-recorded vocal message “Pay attention to your eyes”, meant to facilitate those participants who might have not yet understood the correct origin of the beeps. In the “Inflating” Condition a beep was generated whenever a new ball entered the display (i.e., every 0.3 s starting at display onset until the number of balls for any given trial was reached). In the “Hemifield” Condition the beeps were generated at a fixed rate of 4 Hz, but only when the observer’s gaze was directed to the right hemi-field. No beeps were generated when observers’ gaze was directed to the left hemi-field. We selected the right hemi-field for beeping because observers spontaneously tended to watch the left side of the screen where the balls entered, therefore they could hear the beeps at a constant rhythm for the entire duration of the trial, and guessing their true origin would have been harder. In the “Motion” Condition the beeps were generated at a rate which depended on the instantaneous average velocity of the balls (range: 0.67–4.76 Hz), computed by means of a motion analysis algorithm [39]; **Fig 1B**). These four conditions provided an extended contingency scenario which included two external (Inflating and Motion Conditions) and two internal (Saccades and Hemifield Conditions) causes, and were intended to distinguish the observers’ general capability to discover relations among events from the capability to discover gaze agency.

We opted for a fixed temporal sequence alternating the presentation of an external and an internal beeps’ cause over time. Based on a pilot experiment, we divided our subjects in two groups to which two different condition sequences were administered: “Hemifield → Inflating → Saccades → Motion” for Group 1; “Saccades → Motion → Hemifield → Inflating” for Group 2. In this way the Saccade Condition was either preceded or not by the exposure to the other gaze contingent condition (Hemifield). No counter-balancing was provided.

The two groups (N = 15) were formed by subjects randomly selected from the main group. Participants were not given advance information as to the possible origin of the beeps, and no feedback was given trial-wise. However, at the end of each condition the experimenter revealed the origin of the beeps, regardless of whether or not participants had already guessed correctly the response. In this way, all participants started a new condition with the same baseline information. The Discovery Task lasted about 15 minutes.

#### Detection Task

This task was administered after the Discovery Task, in the same experimental session. The task consisted of 40 trials, each lasting 10 seconds. Visual and auditory stimuli were basically the same as in the Discovery Task, but the visual stimulus comprised always 10 balls, whose motion configuration was varied randomly across trials. In 50% of the trials, randomly interleaved, the beeps were driven by the observer’s saccades. At variance with the Discovery Task, the velocity threshold for saccade detection was lowered to 60 deg/s, so that also small saccades could trigger a beep. In this way, we avoided that the task became too easy: indeed, the instruction was to distinguish between eye movements-generated and externally-generated beeps, thus attention can be presumed to be constantly inward-directed, in the attempt to catch even subtle signs of saccade-contingent–or non-contingent–beeps. Occasional beeps triggered by small saccades should at least slightly increase the task difficulty. In the remaining 50% of the trials the beep sequence was equal to that produced by the observer during a preceding trial, randomly chosen among the N-1 previous trials. That way we ensured that the beep sequences in the two type of trials—contingent and non-contingent–were not too different. At the end of each trial observers had to report whether the beeps were generated by their eyes by pressing a key. A response confidence rating (range 1–5) was also given, again with a key-press. In order to avoid that the task became too easy, observers were invited to watch the balls as naturally as possible, without applying voluntary strategies to verify the origin of the beeps (i.e., close the eyes, keep the eyes still, moving the eyes regularly etc.). The Detection Task lasted about 15 minutes.

### Eye tracking and gaze-contingency

Two-dimensional eye movements were monocularly recorded through infrared oculometry (Dr. Bouis Oculometer limbus tracker, nominal precision <0.3 deg). Before the beginning of each condition in the Discovery Task, and before the beginning of the Detection Task, a 5-points calibration was performed. The analog eye position signals were visualized in real time on an oscilloscope, sampled through an A/D converter (resolution: 12 bit, sampling rate: 600 Hz), and low-pass filtered (300 Hz). Gaze contingency was obtained by computing in real time the eye instantaneous tangential velocity. A beep was triggered when eye velocity exceeded the velocity threshold, provided the inter-beep interval was at least 200 ms (“refractory period”). For technical reasons, the same software loop controlled both the visual display and the sound card drivers, thus the actual temporal resolution for gaze contingency was 60 Hz.

### Gaze contingency

**Fig 2:**
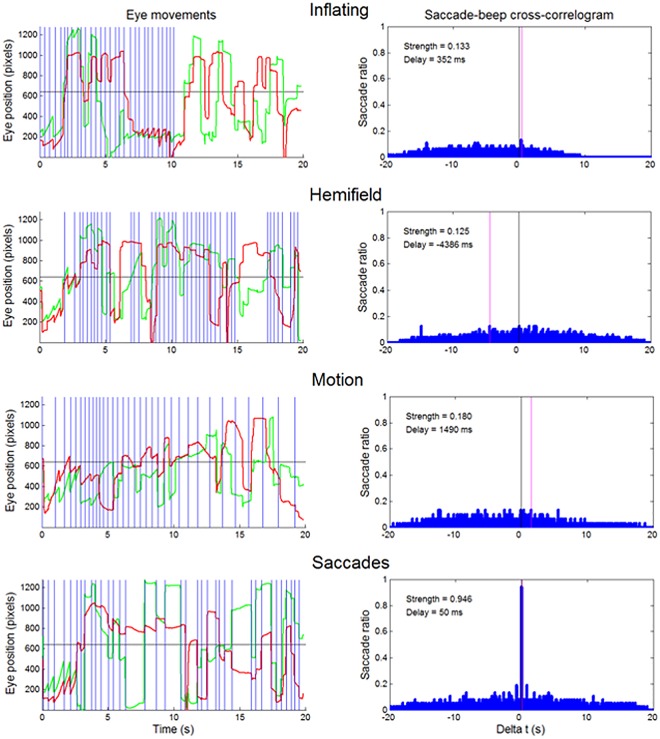
Examples of the temporal relation between saccades and beeps in single trials. Left panels, eye movement traces (green traces: horizontal component of eye position, red traces: vertical component) and beep occurrence (blue vertical lines). Right panels, saccade-beep cross-correlograms. The vertical black and magenta lines indicate zero-delay and the actual delay, respectively.

To quantify the saccade-beep contingency, we applied a cross-correlation analysis to the temporal series of beeps and saccades, both marked by their onset times (**Fig 2**, left panels). The onset times were defined as the moment the beep was triggered on the audio-board, and the moment the instantaneous tangential eye velocity of the eye exceeded the threshold value, respectively. We computed the delay and strength of the temporal contingency between the observer’s saccades and the beeps. These two quantities were computed trial-wise as the horizontal shift of the cross-correlogram peak (**Fig 2**, right panels), and as the ratio of the saccade-beep coincidences (binwidth: 17 ms) relative to the number of saccades, respectively. In the Saccades Condition the strength was on average very high (92% ± 3) and the delay quite low and constant (always 3 video-frames, 50 ms). The strength varied very little across trials (range: 87–94%). By contrast, as expected, in the other three conditions the mean contingency strength between beeps and saccades was very low (on average less than 10%) and without a clear peak (thus the delay was only nominal).

### Data analysis

In the Discovery Task, the performance was quantified on the basis of the raw subjective and objective responses (see the Results section). In the Detection Task, the performance was quantified by means of standard signal detection indexes [40], with a correction for extreme values [41]

As for the ocular behavior, we used the following criteria for off-line eye movement classification [42]: saccades (tangential velocity threshold: 120 deg/s for the Discovery Task and 60 deg/s for the Detection Task; amplitude range: 0.2–40 deg; duration range: 10–200 ms); fixations (dispersion algorithm, minimum displacement: 0.67 deg; minimum duration: 70 ms); smooth pursuit eye movements (velocity range: 2–20 deg/s, duration range: 200–5000 ms).

Data were statistically analyzed by means of Student’s t-test (Discovery Task: z-transformed performance index; Detection Task: sensitivity and bias indexes), mixed-factor ANOVA (oculomotor parameters in the Discovery Task) and ANOVA for repeated measures (oculomotor parameters in the Detection Task). Corrections for multiple comparisons were applied. Distribution’s normality was checked with the Shapiro-Wilk test.

## Results

### Discovery Task

The participants’ responses were coded by 2 experimenters independently, in a 3-way scheme: wrong response, correct response, and quasi-correct response. Examples of the latter category are “the beeps occurred when I was watching the top right part of the screen” (considered to be quasi-correct for Condition Hemifield because of the correct reference to the gaze direction, but incomplete), or “the beeps were generated in the period when the balls entered the display” (considered to be quasi-correct for Condition Inflating despite being generic–the period of entrance of the balls instead of a beep for each entrance).

**Fig 3:**
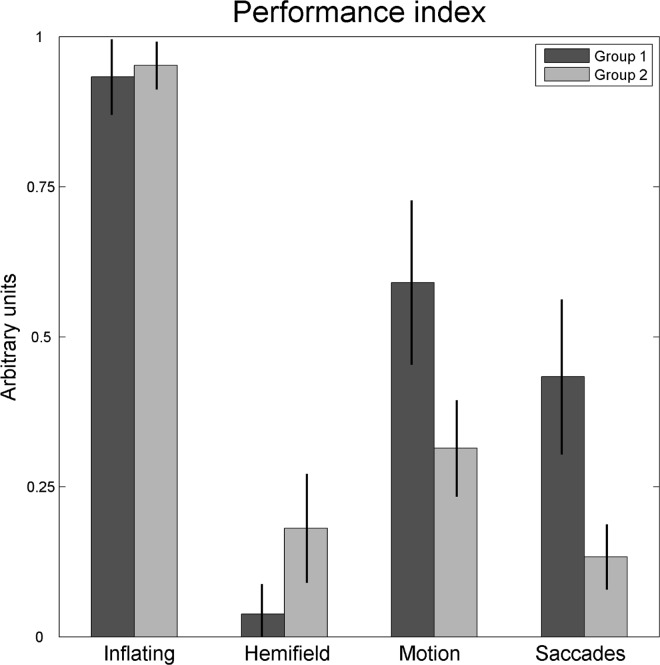
Mean values and 99% confidence intervals of the Performance Index in the four conditions of the Discovery Task.

**Fig 4:**
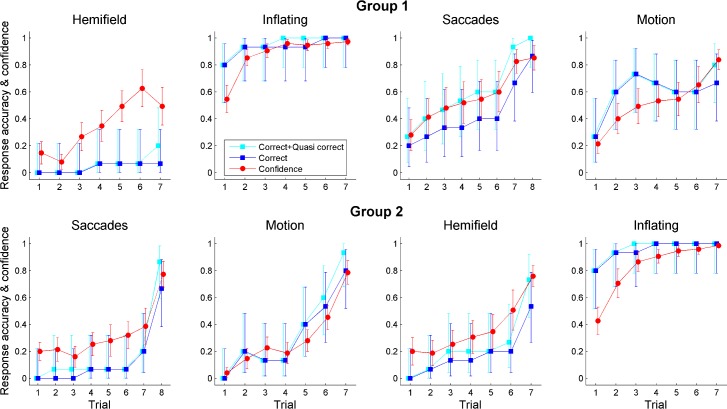
Time-course of task performance over successive trials. The blue and cyan data-points represent the objective performance (accuracy, correct and quasi-correct responses respectively) while the red data-points represent the subjective performance (confidence, normalized). The left-to-right order of conditions represents the real temporal sequence of presentation in the two groups (Conditions’ sequence for Group 1: “Hemifield → Inflating → Saccades → Motion”; for Group 2: “Saccades → Motion → Hemifield → Inflating”). Error bars represent 99% confidence intervals of the means.

For each subject and for each condition we computed the ratio between the number of correct responses (cr) over trials and the number of trials (nt), and called this quantity Performance Index ($\frac{\sum_{1}^{nt}cr}{nt}$). Under the assumption of a monotonic increase over trials, the Performance Index increases with the speed of the observer’s discovery of the correct origin of the beeps: if the solution in the Saccade Condition is found in the first trial the index is 1, while if it is found in the fifth trial, the index is 0.5. The index is 0 if the solution is never found. The results are shown in **Fig 3**, separately for each group and condition. We chose to calculate this Performance Index in order to obtain a single value conveying the discovery phenomenon, although at the cost of some information loss (e.g., temporal dynamics reported in **Fig 4**).

The pattern of results of Group 1 was quite remarkable, as we achieved two conditions, one internal and one external, with an intermediate discovery performance (Saccades and Motion), and two conditions, again one internal and one external, in which the performance tended to saturate, either at ceiling or at floor (Inflating and Hemifield, respectively, where the performance index was not significantly different from one and zero, t(14) = 1.469, p = 0.918, and t(14) = 3.327, p = 0.998, respectively). Because despite the transformation not all distributions fit the normality assumption, we further run a non-parametric median test, which confirmed the above results (Wilcoxon signed-rank test, p>0.125 for both Inflating and Hemifield Conditions). By contrast, the pattern of results of Group 2 turned out to be less balanced, and, notably, in the Saccades Condition the performance was quite low. Except for the Inflating Condition, the task performance depended on the order of conditions presentation: as compared to Group 1, the performance of Group 2 decreased significantly in the Saccade Conditions (-20%, t(28) = 2.558, p = 0.008), and increased in the Hemifield Condition (+14%, t(28) = -2.780, p = 0.005). The median test confirmed these results (p = 0.021 and p = 0.016, respectively for the Saccade and Hemifield Conditions).

We then plotted the proportion of correct responses (accuracy) as a function of the progressive trial number, together with the mean confidence rating (**Fig 4**). The observers’ capability to discover the origin of the beeps tended to increase with trial number. In the crucial Saccades Condition, the mean performance index of Group 1 observers was 0.43 ± 0.13 S.D. (**Fig 3**), with an accuracy varying between 0.20 and 0.87 when passing from the first to the last trial (**Fig 4**). We recall that this performance was obtained after a previous exposure to gaze-contingency (Hemifield Condition), which suggests that discovering visual agency is not an easy task. Indeed, even after the verbal suggestion there were still two participants who did not find the correct solution (though they provided a quasi-correct answer). In Group 2, the effect of the verbal hint was to push discovery accuracy from 20% to 67%. By comparison, the effect of the hint on the observers of the Group 1, who were already exposed to an instance of gaze-contingent beeping, was much weaker (from 67% to 87%). This was even more evident when considering also quasi-correct responses (Group 2: from 20% to 87%; Group 1: from 93% to 100%).

In the Motion Condition, where the beeps were generated by an external factor, the accuracy range in Group 1 was 0.27–0.67 over trials, though with a rapid rise that generated a peak of accuracy at the third trial, followed by a moderate inflexion in the following trials. The mean performance index was 0.59 (±0.21 S.D.). In Group 2, the mean performance index was 0.31 (±0.12 S.D.), with an accuracy range between 0 and 0.8 over trials.

By contrast, in the Inflating Condition the performance index was almost saturating (0.93 ±0.09 S.D.), while in the Hemifield Condition it was close to zero (0.04 ±0.07 S.D.). Correspondingly, the accuracy ranges were 0.80–1 and 0–0.067, respectively. In Group 2, the performance index was 0.95 ±0.06 S.D. in the Inflating Condition and 0.18 ±0.13 S.D. in the Hemifield Condition (accuracy ranging between 0.80–1 and 0–0.53, respectively). Being almost at ceiling and floor, these two tasks could thus reveal only one-directional performance changes.

As to the quasi-correct responses, they contributed to improve systematically the performance especially in the Saccades Condition, which suggests that a number of observers were in fact not too far from understanding that their saccades were causing the beeps. The performance when correct and quasi-correct responses are considered together is shown in **S1 Fig**, for all conditions in both groups.

The pattern of results for the confidence ratings was qualitatively similar to the accuracy pattern, in that both tended to increase with decreasing ball number (**Fig 4**), though with less variation across conditions, especially the Inflating and Hemifield Conditions (**S2 Fig**). This suggests that the self-confidence observers was less influenced by the different conditions and their order, than revealed by objective accuracy.

In order to find the solution, observers should monitor, implicitly or explicitly, the temporal contingency between the beeps and the candidate events. In particular, in the Saccades Condition observers had to understand that the beeps were generated by their own saccadic eye movements. On average, in each trial there were 32, 10, 29 and 17 beeps, respectively for the Hemifield, Inflating, Saccades and Motion Conditions (means of the 2 Groups). In both the Hemifield and the Saccade Conditions there were more beeps in Group 2 (30 vs. 34, t(28) = 2.239, p = 0.033, and 27 vs. 31, t(28) = 2.759, p = 0.010, respectively). In the other two Conditions beeping was identical in the two Groups because beeps were generated by the visual display.

**Fig 5:**
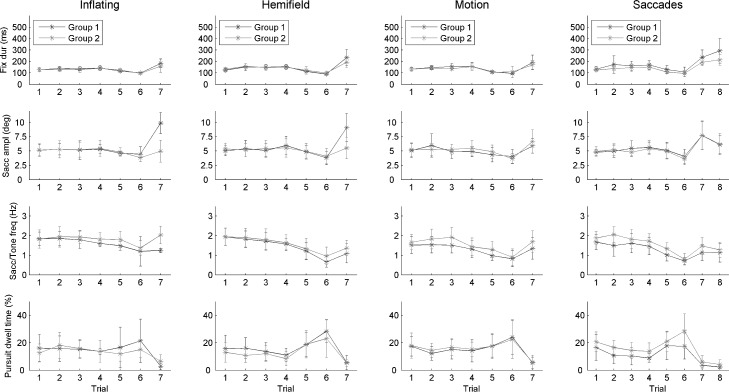
Time-course of the four oculomotor parameters over successive trials in the Discovery Task, plotted separately for each Condition (Hemifield, Inflating, Saccades, Motion). Bars represent standard deviations. The non-null smooth pursuit dwell time values detected when the balls are stationary (7th and 8th trials) are likely due to residual slow eye motion–e.g., post-saccadic drift–and/or noise. The two observer groups are identified by separate lines in each plot.

We then characterized the observers’ oculomotor activity. Indeed, the ocular behavior may have influenced the capability of discovering the cause of the beeps. The analysis of eye movements was based on four oculomotor parameters, namely, fixation duration, saccade amplitude, saccade frequency and time spent in smooth pursuit (dwell time). These parameters are shown in **Fig 5** as a function of group and trial number, separately for the four different Conditions.

For each parameter, the pattern of results was remarkably similar across the two groups and the four experimental conditions, with a systematic, highly significant main effect of trial in each of the 16 plots (always p<0.001). These data suggest that the observers’ gaze was largely dictated by the visual configuration of the visual stimulus–which was identical among the conditions but different along the trials. In particular, there was a marked contrast between the trials in which the balls were moving (1^st^-6^th^) and the trials in which the balls were stationary (7^th^ and 8^th^), which may correspond to a different balance between saccades and smooth pursuit eye movements. There were no oculomotor differences due to the different exposure sequence in Group 1 and Group 2 (lack of a significant main effect of Group or interaction Group x Trial, with the exceptions of a main effect of Group for smooth pursuit dwell time, saccade frequency and fixation duration in the Saccades condition, where Group 2 observers were more engaged in smooth pursuit eye movements than Group 1 observers, F(1,28) = 16.945, p<0.001, and showed a higher saccade frequency (F(1,28) = 11.044, p = 0.002) and shorter fixation durations, F(1,28) = 14.671, p = 0.001; these differences were on average rather modest, as smooth pursuit dwell time passed from 10.9% to 15.6%, saccade frequency passed from 1.3/s to 1.5/s, and fixation duration passed from 175 to 146 ms. Because the fixation duration distributions did not fit the normality assumption, we validated the previous analysis with logarithmic data transformation (data not shown). In addition to the mean, we analyzed also the variability of fixation duration, computed as the variance of gaze position over time, which could be another sensitive index of a change in visual exploration style, but neither the main effect of Group nor the interaction Group x Trial reached statistical significance. Thus, it appears that observers did not find the solution by modulating their visual exploratory activity, at least in a macroscopic way.

### Detection Task

In this task observers had to explicitly report whether or not the beeps were generated by their own eye movements. This task implied a forced-choice discrimination between a contingent and a non-contingent condition. The detection performance was quantified by means of accuracy and confidence measures, as well as signal detection indexes (d’ and logβ) [40]. The mean accuracy, i.e., the proportion of correct responses, was rather high (77% ± 14%), with a mean confidence of 0.67±0.18 (normalized values in the 0–1 range). In terms of signal detection performance, observers showed a fairly good sensitivity for saccade-contingent beeps, with a mean d’ of 1.83±1.20, which was significantly higher than zero (t(29) = 8.314, p<0.001), and with a mean negative bias which however was not significantly different from zero (-0.20±0.83, t(29) = 1.336, p = 0.192). These results are reported in **Table 1**.

**Table 1:** Detection Task performance.

	Accuracy	Confidence	d'	log β
*Mean*	0.773	0.669	1.827	-0.201
*S*.*D*.	0.138	0.184	1.203	0.825

Thus, also this task affords both positive and negative variation measurements, which can assess the effect of experimental manipulations resulting in either a sensitivity increase or decrease.

**Fig 6:**
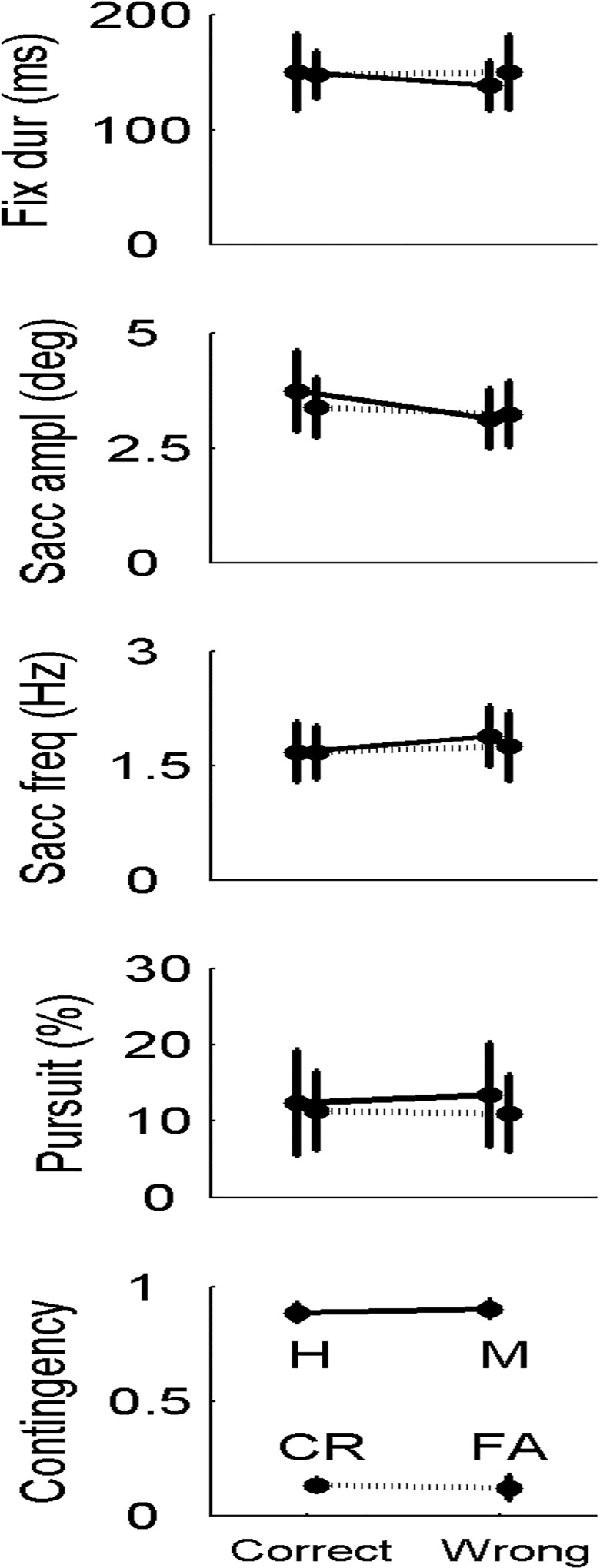
Oculomotor behavior (top 4 panels) and contingency strength (bottom panel) in the Detection Task. The results are grouped in terms of stimulus category (contingent vs. non-contingent trials, different lines, same for all panels) and response type (correct vs. wrong responses, horizontal axis, same for all panels). Bars represent the standard deviation. H = Hits, M = Misses, CR = Correct Rejections, FA = False Alarms.

We asked whether the oculomotor behavior could have been at the origin of the observers’ capability to find the correct answer. For example, observers might have voluntarily modulated their eye movements to detect the presence of beep-saccade contingency, despite the explicit instruction given by the experimenters before the beginning of the task. In **Fig 6** are reported the results for the four parameters already used to quantify the oculomotor behavior in the Discovery Task (i.e., fixation duration, saccade amplitude, saccade frequency and smooth pursuit dwell time), as well as the contingency strength.

The results are shown separately for hit trials (response ‘contingent’ | contingent beeps), correct rejection trials (response ‘non contingent’ | non-contingent beeps), miss trials (response ‘non contingent’ | contingent beeps) and false alarm trials (response ‘contingent’ | non-contingent beeps). This allowed us to search for possible associations between the task performance and eye movements in a trial-wise way. Overall, both the oculomotor behavior and the contingency strength were rather similar to the Discovery Task, although a direct comparison cannot be performed due to the differences between the two tasks. At a first sight, none of the four oculomotor parameters seemed to be related to the task performance. However, correct responses were associated to small but significant decreases of saccade frequency (main effect of response type: F(1,29) = 18.539, p<0.001) and amplitude (main effect of response type: F(1,29) = 14.031, p<0.001). The saccade-beep contingency strength (**Fig 6**, bottom panel) clearly reflected the stimulus category (main effect of stimulus category: F(1,29) = 7042.899, p<0.001), and not the response type (main effect of response type: F(1,29) = 0.042, p = 0.840). The same pattern was obtained by computing the contingency strength over a looser 200 ms time window (data not shown).

## Discussion

In this study we have introduced two tasks aimed at assessing the sense of self-agency exerted through eye movements, which we have called gaze agency. Both tasks required observers to recognize the causal relation between their own eye movements and audio-visual contingencies, but one task was aimed to measure the capability of spontaneously discovering gaze agency (propensity to self-monitoring), while the other task was aimed to measure agency detection sensitivity. Our approach combined a classical psychophysical task (Detection Task) with a task intended to mimic real-life circumstances (Discovery Task). Indeed, in ordinary life we are rarely required to explicitly discriminate the exogenous vs. endogenous origin of an event; rather, we tend to link causes and effects without explicit constraints, much like in our Discovery Task.

### Detection Task

The Detection Task involved a psychophysical design within the framework of signal detection theory [40], which included a very common “currency” to measure observers’ sensitivity (d’). As such, this task is analogous to tasks previously used to assess bodily agency, which share a similar experimental logic (e.g., finger tapping agency detection, [17]). In probing a condition in which observers are already focused on gaze agency–and not, as in the Discovery Task, on finding a relation between events–the solution space in the Detection Task is much narrower than in the Discovery Task, thus making this task ideal to measure the sensitivity to visuo-motor contingency. The fact that the observers’ performance settled down at intermediate levels, and that no significant bias was found, indicates that the current stimulus/task parameters are well tailored for assessing both increases and decreases of gaze agency.

The observers’ performance could depend on small variations of eye movements pattern. However, the only statistically significant oculomotor variation associated to wrong responses in the Detection Task was a small increase in saccade frequency, accompanied by a small decrease of saccade amplitude. While in principle a higher saccade frequency might be more confounding for the observer (more events to monitor), in our data such increase was in fact very modest (corresponding to a difference of only 1 saccade over the 10-s trial duration: on average 17 versus 16 saccades respectively in wrong and correct trials), thus it is not obvious how this little difference could explain wrong trials. A similar argument holds for saccadic amplitude. Thus, we found a dissociation between performance and eye movements. This observation makes it unlikely that an explicit, macroscopic oculomotor strategy was applied to solve the task, in spite of the fact that in this task–at variance with the Discovery Task–observers were well aware that their goal was precisely to detect eye movement-contingent beeps. The instruction to behave as naturally as possible may have contributed to this dissociation.

The observers’ performance could depend on changes in the saccade-beep contingency. In principle, for example, misses and false alarms might occur following a decrease and an increase of the contingency strength in contingent and non-contingent trials, respectively; however, in contingent trials the saccade-beep contingency was fixed experimentally, and in non-contingent trials the contingent strength was not higher with false alarms than with correct rejections.

### Discovery Task

The structure of the Discovery Task was very different from that of the Detection Task. Here, observers were not aware of the specific goal of the task, i.e., they were not told in advance that gaze agency was at stake. Thus, this task was well suited to probe the tendency to orient attention to one’s own eye movements, which in turn may depend on the propensity to orient attention to oneself (self-awareness).

The four conditions used in this task were not intended to be contrasted one against the other, as they represent different experimental conditions sharing a number of elements but in fact qualitatively dissimilar. Rather, each condition was intended to be contrasted with the same condition across different experimental groups, e.g., patients vs. healthy observers, children vs adults, or treatment vs. control. As a test-bed, we compared the task performance in two groups of observers in which a different condition sequence was administered. We did not attempt to counterbalance the temporal order presentation, which would have required many more groups, as we were primarily interested to verify if a noticeable performance change would have occurred at all. Note however that this was not a validation study, which would have required a much larger sample.

The main findings were an increase of the Performance index in the Saccades Condition when observers were previously exposed to another instance of gaze contingency (Hemifield Condition), and a specular increase in the Hemifield Condition after exposure to the Saccades Condition. The Inflating Condition was probably too easy, as observers were almost always correct. A performance increase in Group 1 vs. Group 2 was also observed in the Motion Condition, though it did not reach statistical significance after correction for multiple comparisons. Because it is difficult to fully exclude that such increase is in fact due to a systematic effect, we cautionary consider it as a possible general performance improvement due to learning or exposure. Another finding was that the hint, through which observers were invited to pay attention to their eyes, dramatically improved the performance of Group 2 observers, who were not yet previously exposed to an instance of gaze contingency, but much less so in Group 1, whose discovery performance was already rather high.

The better performance in the Saccades Condition found in Group 1 as compared to Group 2 is arguably due to the previous exposure to a gaze-contingent condition (Hemifield Condition), which made the observers aware of the possibility that gaze direction may affect beeping. However, given the above-mentioned possibility of a general effect of exposure or learning, this conclusion should be paired with the observation that the hint improved the performance in Group 2. Thus, manipulating self-awareness resulted in a clear performance increase within-group (effect of the hint), and probably also between-groups (previous exposure to gaze contingency).

These results suggest also that the Group 1 sequence is better suited to probe agency at the group level, as compared to the Group 2 sequence. Ideally, a perfectly calibrated system should provide, with healthy subjects, a performance index of 0.5 with both an internal and an external condition (the performance index has a 0–1 range). This way, one can measure equally well self-agency increases and decrease, as well as other-agency increases and decreases. The values of performance index measured in the Saccades and the Motion Conditions in Group 1 subjects were close to such an ideal situation. This is relevant in the perspective of applying this tool/method to study clinical and developmental populations that may show either increases or decreases of the sense of agency (see below).

We sought to gauge the possible different strategies adopted by the observers to solve the tasks by analyzing their eye movements. Indeed, it is well known that top-down control of eye movements may reveal the underlying cognitive schemes [43,44,45,46]. Thus, detecting the saccade-beep contingency might be accompanied by a distinctive oculomotor behavior, perhaps demarcating the transition from exploratory to performative eye movements (note that this is the opposite of what happens with the hands, when the transition is rather from performative to exploratory function [47]). However, in the Discovery Task the oculomotor behavior was unrelated to the observers’ accuracy level, but rather to the display configuration, which varied by passing from the first to the last trial. Also, we found only minor oculomotor differences between Group 1 and Group 2, which rules out important strategic changes in visual exploration as a result of previous experience. This seems to leave little room for learning processes, even more so because when Group 1 observers started the Saccades Condition they were aware of the possibility that the gaze could be the cause of the beeps. We have no obvious answer as to the reasons of this dissociation between task performance and oculomotor behavior, but given the body of evidence showing the effect of expertise on eye movements (see [48,49]) it is possible that the oculomotor behavior changes only after a long-term, specific training.

### Contingency detection

It appears that in both Discovery and Detection Tasks the solution emerged out of an almost steady background oculomotor activity, in which understanding the correct cause of the beeps escaped the statistics of eye movements. Unfortunately, we do not know the exact moment, within a trial, in which observers opted for a given response. It is possible that local oculomotor variations involving very few saccades/beeps could reveal subtle strategies–or simply fortunate random fluctuations–of visual inspection associated to correct decision-making.

As for the possible underlying mechanisms, gaze agency might not entail the monitoring of a neural oculomotor component (efference copy), but the visual or proprioceptive re-afferent signals contingent to eye movements, which would imply a sensori-sensory rather than sensori-motor contingency detection mechanism. Gaze agency might also depend on a contingency mechanism based on visuospatial attention or even on the mere intention to move the eyes. In principle, these different contingency mechanisms should be tuned to different temporal relations between the relevant events. For example, detecting a saccade-beep contingency is expected to take longer if it is based on visual re-afference than if it is based on efference copy or attention signals, because the former is subjected to visual delays [50,51] while the latter are internal signals that co-occur or even precede a saccade [52].

Although from our data we cannot tell which precise contingency detection mechanism is gaze agency based on, the results concerning the Hemifield and the Saccades Conditions in the Discovery Task seem to suggest that we are more prone to monitor internal events than the visual consequences of eye movements. In fact, the Hemifield Condition, in which the correct solution is

supposed to involve the detection of a visual fact (‘I am looking there’), turned out to be more difficult than the Saccades Condition, in which the correct solution is supposed to require only internal monitoring (‘I turned my eyes’) because of the visual stability across saccades [53]. This may seem surprising because in the first place eye movements imply a visual rather than a motor phenomenology. A simpler account is that it is easier to match single events (pairing single saccades and single beeps) than states (‘I am looking there’ paired with a beep sequence). The latter interpretation is also suggested by the high performance in the Inflating condition (‘one bubble one beep’).

### Gaze agency

Gaze-operated and attention-aware devices are going to become a pervasive presence in our environments [5,6,7,54], and it is therefore likely that gaze actions will soon become part of our cognitive repertoire. Besides the advantages associated to this new interaction opportunity, however, endowing the gaze with a performative function bears observers with the novel responsibility of considering the physical consequences of their eye movements (think at the consequences of, say, forgetting the very possibility of changing the physical/technological world with the gaze, or of being trapped in the “Mida’s touch” problem). It might even be that the performative function comes at the cost of the natural exploratory function, hence ultimately changing or even worsening vision. For example, certain visual objects may acquire a new affordance (‘look at me’, [55]). Also, adding this new function to the oculomotor repertoire might imply that observers are more often engaged in multi-tasking [56]. Thus, we deemed it important to open a window into gaze agency, starting by providing an experimental setting that allows to measure it.

As already mentioned in the Introduction, the sense of agency is commonly assessed by instructing observers to produce a given bodily movement and to judge its perceptual consequences [30]. However, in many studies reported in the literature [9,57](9–33), participants were aware that agency was the probed capacity, that is, their attentional stance was already clearly oriented. This precludes the possibility to measure the propensity to spontaneously orienting attention towards oneself rather than towards the external world. Yet, this may be an important aspect of agency, especially in neurodevelopmental and pathological conditions (see below). By combining the Detection and the Discovery Task, our approach overcomes this limitation, and may reveal both cognitive aspects of agency attribution and low-level aspects of sensori-motor contingency detection. For example, troubles with self-awareness may not affect the latter, but only the tendency to orient attention along the internal/external axis. Indeed, the two simple awareness manipulations that we applied in the present study, i.e., previous exposure to gaze contingency and verbal hint, were expected to modify the performance in the Discovery but obviously not in the Detection Task.

An advantage of using eye movements to probe high-level aspects of agency attribution is that they provide a continuous and natural source of movement. This relieves from the need to assign a specific motor task to the observers, even a simple one, which would change *ipso facto* their attentional stance. Granted, there are other bodily movements that just “occur” (i.e., without the need of an explicit instruction), and which in fact have been exploited to probe the emergence of the sense of agency, namely, infants’ sucking and leg kicking movements [58,59]. Other bodily movements that could be likewise exploited to probe agency are blinking and breathing. Indeed, blinking has been used successfully in human-computer interfaces [54]. As to breathing, paying attention to this peculiar movement involves interoceptive neural mechanisms [34], whereas gaze agency involve a combination of proprioceptive and exteroceptive sensing (see above). Thus, breathing might be better suited to investigate self-awareness based on viscerosomatic and emotional sensations. Moreover, compared to eye movements blinking and breathing have a limited performative capacity: for example, they are not spatial pointers.

At variance with bodily agency, gaze agency entails a remote–and not a mechanical–action, in the sense that cause and effect are not mechanically coupled but configure an action at distance. The fact that an object can be acted upon without physical contact confers a special status to gaze agency. As such, gaze agency is well suited to probe cognitive capabilities that span from action monitoring [22] to social functioning [27]. It would be interesting to directly compare the sense of agency when a movement acts mechanically (bodily movements) or remotely (eye movements). For example, it appears that infants are very good at forming artificial sensori-motor associations, perhaps because, differently than adults, they “start from scratch” [60].

Gaze agency in the physical/technological domain is different from social gaze agency. Indeed, although both rely on an action at distance, social agency has a communicative function, whereas physical agency has only a performative function, such as for example button switching in gaze-operated devices. However, there is a growing literature on the different effects of saccadic eye movements suggesting that similar associative and predictive mechanisms may govern the acquisition and usage of social and non-social effects of eye movements [4,23,24,61]. It is therefore entirely possible that, similarly to other behaviors and traits (e.g., [62,63]), also gaze agency can take advantage of existing neural mechanisms to implement a new function. For example, a flexible contingency detection mechanism sensitive to both loose and strict action-response coupling could implement both forms of gaze agency [64]. Yet, it is presumable that non-social agency would not be accompanied by other overt communicative signs such as facial expressions, gestures or body postures. This is an interesting issue to address in future research.

### Defective agency

Distinguishing inner from outer facts is a key capability for the correct development or maintenance of a reality-based stance. Indeed, in some psychiatric and neurodegenerative diseases the exogenous-endogenous distinction is known or suspected to be altered. The process of self-recognition/self-monitoring could be split in two levels: an automatic level for action identification and a conscious level for the sense of agency [28,30,65,66]. This second level, which comprises intentions, plans and desirers, is damaged in schizophrenic patients, who fail to ascribe their internal speech, thoughts, covert and overt actions to themselves. A misattribution of actions’ effects may be also involved in patients with Alzheimer’s disease, which substitute causal explanations of their actions’ consequences with teleological ones [67]. This is reminiscent of children’s explanation of phenomena referring to their function [68]. By contrast, patients affected by Obsessive Compulsive Disorder (OCD) may show an altered awareness of action at the pre-reflective/automatic level (first level, [69]). An altered sense of agency was also found in Parkinson Disease patients on medication, probably due to an increased action-effect binding [70]. In all these cases, our approach could extend agency testing by probing how patients discover a novel agency capability. By tapping not only on sensori-motor capabilities but also on the attentional stance, probing gaze agency seems to target reasonably well also the second level of self-recognition/self-monitoring.

Attributing an external or an internal cause to a given event or phenomenon may depend on how much we tend to allocate attention towards the external or the internal world [71]. For example, a poor capability to spontaneously discovering gaze agency may be associated to an exaggerated outward-oriented attention allocation. In evaluating the propensity to find internal vs external causes of events, our approach may deepen our knowledge also of those neurodevelopmental disorders where attentional control is defective (e.g., Autism Spectrum Disorders, [72]; Attention Deficit/Hyperactivity Disorder, [73]). Indeed, a testing tool that probes not only agency sensitivity but also agency propensity may be better suited to discover subtle agency dysfunctions related to attentional unbalance that may go unnoticed with traditional tests [20,74,75,76], and could be also useful to clarify conflicting data reported in the literature [77,78,79]. Additionally, gaze agency might be useful to investigate more generally the emergence and timeline development of the sense of agency in childhood [80,81,82].

## Conclusions

In summary, the proposed method proved useful to assess gaze agency, both in terms of spontaneous discovery and detection capability. Compared to previous studies, our method addresses a very special sense of agency, involving remote causal action of eye movements on the physical/technological world, and taps on both sensori-motor processing and cognitive capabilities. As a test-bed, we compared two groups of observers to whom different condition sequences were administered, and found an effect of learning or exposure, which however did not translate into a macroscopic change of the oculomotor pattern. A large performance increase was found after the hint in which observers were invited to pay attention to their eyes, which showed that orienting attention to oneself, i.e., increasing self-awareness, modifies the sense of agency. Candidate populations to be probed for gaze agency are patients with known or suspected defective sense of agency. Young children could also be screened for a possible early diagnosis of autism or ADHD and to study the development of the sense of agency.

This research was supported by the GRANT “Futuro in ricerca 2013” (http://firb.miur.it/ grant number: RBFR1332DJ) from the Italian Ministry of Education, Universities and Research.

## Supporting Information

S1 Data S1 Data is an Excel file containing data on subject responses and confidence of the Discovery Task: it contains a single 5-dimensional numerical array: Data Out (2,15,4,8,2), Dimension 1 codes Group (1:2), Dimension 2 codes Subject (1:15), Dimension 3 codes Condition (1:4), Dimension 4 codes Trial (1:8). Dimension 5 contains measured values (1 = Response, 2 = Confidence).
(XLSX)

S2 Data S2 Data is an Excel file with data on subject eye movements of the Discovery Task: it contains a single 4-dimensional structure with 4 numerical fields, each containing the mean value in that trial for the specified oculomotor parameter: DataOut = 4-D struct array with fields: Fixdur (fixation duration), Saccampl (saccade amplitude), Saccfr (saccade frequency), SmoothDwell (smooth pursuit dwell time). → Fixdur (fixation duration). → Saccampl (saccade amplitude). → Saccfr (saccade frequency). → SmoothDwell (smooth pursuit dwell time). The dimensions are: DataOut(15,2,4,8).field. Dimension 1 codes Subject (1:15), Dimension 2 codes Group (1:2), Dimension 3 codes Condition (1:4), Dimension 4 codes Trial (1:8).
(XLSX)

S3 Data S3 Data is an Excel file with data on subject responses and eye movements of the Detect Task: it contains a single 1-dimensional structure with 30 numerical fields, each containing the mean value, over trials, for the specified parameter in each subject: DataOut = → 1x30 struct array with fields: Accuracy (proportion of correct trials), Confidence (confidence rating), Dprime (d’), Bias (bias), StrengthHITS (strength of the cross-correlation in Hit trials), DelayHITS (delay of the cross-correlation in Hit trials), StrengthMISS (*same in Miss trials)*, DelayMISS, StrengthFA (*same in False Alarm trials)*, DelayFA, StrengthCR (*same in Correct Rejection trials*), DelayCR, SaccamplHITS (saccade amplitude in Hit trials), SaccfreqHITS (saccade frequency in Hit trials), FixdurHITS (saccade frequency in Hit trials), SmoothdwellHITS (smooth pursuit dwell time in Hit trials), SaccamplMISS (*same in Miss trials*), SaccfreqMISS, FixdurMISS, SmoothdwellMISS, SaccamplFA (*same in False Alarm trials*), SaccfreqFA, FixdurFA, SmoothdwellFA, SaccamplCR (*same in Correct Rejection trials*), SaccfreqCR, FixdurCR, SmoothdwellCR. (XLSX)

S1 Fig Mean values and 99% confidence intervals of the performance index in the four Conditions of the Discovery task. Correct and quasi-correct responses are pooled together.
(TIF)

S2 Fig Mean values and 99% confidence intervals of confidence ratings in the four Conditions of the Discovery task. (TIF)
