# Supplementary figures and images for: Assessing Self-Awareness through Gaze Agency
Source: PLoS One. 2016 Nov 3;11(11):e0164682. doi: 10.1371/journal.pone.0164682 (PMC5094589; doi:10.1371/journal.pone.0164682)

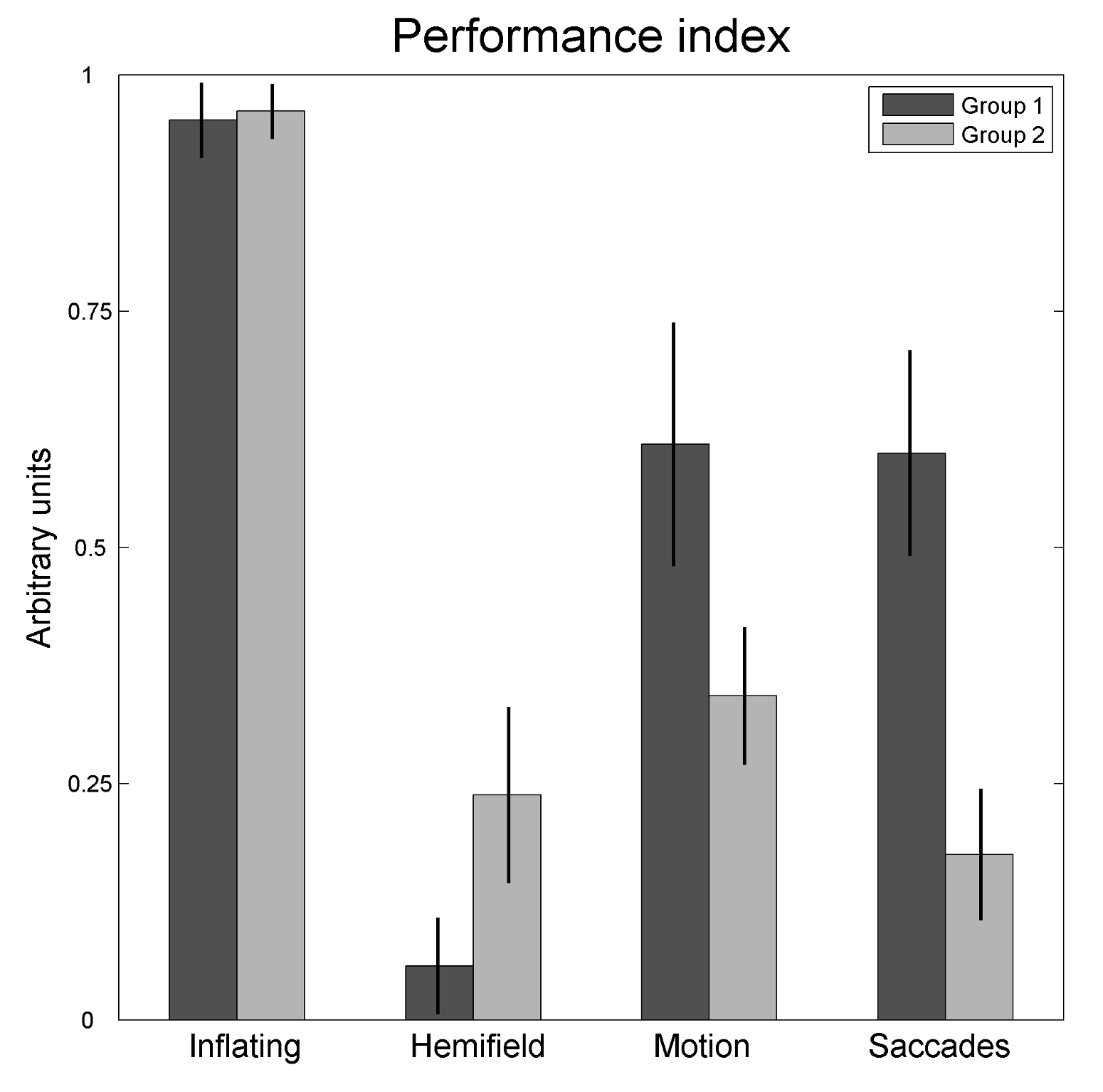

Supplement: S1 Fig — Correct and quasi-correct responses are pooled together. (TIF) [file pone.0164682.s004.tif]

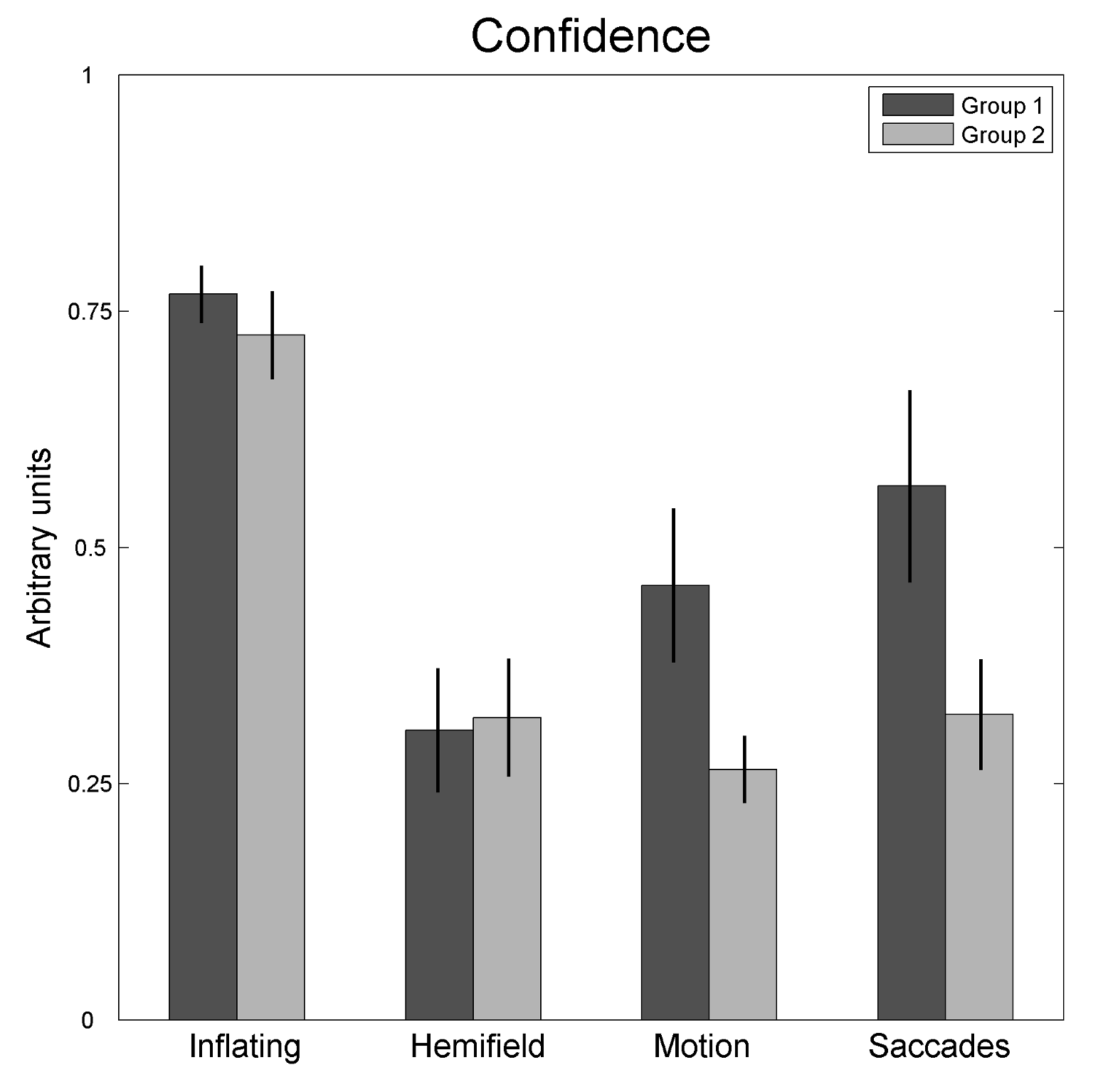

Supplement: S2 Fig — (TIF) [file pone.0164682.s005.tif]
